# Arsenic and Diabetes

**DOI:** 10.1289/ehp.1206100

**Published:** 2013-03-01

**Authors:** Allan H. Smith

**Affiliations:** School of Public Health, University of California, Berkeley, California, E-mail: ahsmith@berkeley.edu

Maull et al. (2012) reviewed evidence linking arsenic with diabetes in an evaluation that I believe could divert research resources from where they should properly be allocated. I wish to make two points:

The review gives credibility to flawed studies that conclude that the prevalence of diabetes is increased in people having urine arsenic concentrations in the upper 20% of the general U.S. population.

The authors implied that we need studies assessing arsenic concentrations < 150 µg/L in drinking water, whereas research should actually focus on 150–500 µg/L.

Regarding the first point, Table 2 of the review by Maull et al. (2012) reported an adjusted odds ratio (OR) of 3.58 for diabetes in the upper quintile of U.S. urinary arsenic concentrations (Navas-Acien et al. 2008). When adjusted for sex, age, race, and creatinine (Navas-Acien et al. 2008), the OR was 0.82, and adjustment for four more factors resulted in an OR of 1.05. Navas-Acien et al. inserted two more variables into the regression model, including arsenobetaine (a nontoxic form of arsenic originating from fish), and the OR jumped up to 3.58. Never in the history of epidemiology have valid findings emerged from results like these. For > 20 years, arsenic researchers have been subtracting arsenobetaine from total arsenic in urine when assessing exposure to inorganic arsenic. When this is done, the OR estimate is 1.15 (Steinmaus et al. 2009a).

If the OR of 3.58 were valid, then very low concentrations of arsenic in water would be a major risk factor for diabetes. Among the 40 million or so adults within the highest quintile of urinary arsenic concentrations in the United States, > 4 million would become diabetic, attributable to low arsenic exposure. However, the OR estimate lacks scientific plausibility, with urine arsenic concentrations in the United States about 10 times lower than those related to diabetes in Taiwan, Bangladesh, and elsewhere, and with U.S. water arsenic concentrations about 50 times lower.

In their Table 2, Maull et al. (2012) also cited another paper by the same authors that claims there are increased risks of diabetes related to arsenic in the United States (Navas-Acien et al. 2009). Again, the OR suddenly jumped up after inappropriately adding variables into the multivariate analysis (Steinmaus et al. 2009b). Yet this review from Maull et al. (2012) presented Navas-Acien et al.’s results as if they were from valid methods of analyzing the data. These analyses should not have been cited or their mistakes should have been acknowledged.

With regard to the point that studies should assess arsenic concentrations 150–500 µg/L in drinking water, there is good evidence that arsenic in water may increase the incidence of diabetes. However, every study that has produced strong evidence has included water arsenic concentrations > 500 µg/L at, or before, the time of the study. Indeed, Maull et al. (2012) cited one large, well-designed study in Bangladesh (Chen et al. 2010) with water arsenic concentrations up to 500 µg/L that found no evidence of increased diabetes, even among the > 2,000 participants with urinary arsenic concentrations > 200 µg/L.

In courts of law, experts may be entitled to their opinions, but in science we are not. We must focus only on the evidence and its logical interpretation. The logical interpretation of the evidence here should lead us to pursue studies in populations exposed to arsenic in drinking water in the range of 150–500 µg/L and to dismiss the notion that millions of people in the United States with very low exposure to arsenic in drinking water have major increased risks of diabetes.

In the past, I was attacked for exaggerating the effects of arsenic in drinking water, including in this journal (Carlson-Lynch et al. 1994). Now I find myself on the other side. In 1995, it was said that epidemiology was facing its limits (Taubes 1995); at that time I thought these criticisms were unfair (Smith 1995). But now epidemiology is going beyond its limits. Limited research resources should focus on biologically plausible, detectable risks, recognizing that protecting the general population which has very low exposure involves extrapolating risks downward from higher exposure studies, and accepting that we may never prove whether risk estimates at very low exposures are real or not.
